# The Effect and Safety of Thunder-Fire Moxibustion for Low Back Pain: A Meta-Analysis of Randomized Controlled Trials

**DOI:** 10.1155/2022/6114417

**Published:** 2022-05-18

**Authors:** Yao Yao, Lin Zhou, Feng-qin Chen, Rui Zhang, Xiang-tian Pang, Yu-fei Leng, Xiao Xu, Zhi-ling Sun

**Affiliations:** ^1^School of Nursing, Nanjing University of Chinese Medicine, Nanjing, Jiangsu Province 210000, China; ^2^Office of Academic Affairs, Nanjing Normal University of Special Education, Nanjing, Jiangsu Province 210038, China; ^3^Auxiliary Teaching Center, Shanghai Jiao Tong University School of Medicine, Shanghai, 200025, China; ^4^School of Nursing, Zhejiang Chinese Medical University, Hangzhou, Zhejiang Province 310053, China

## Abstract

**Background:**

Low back pain (LBP) is considered the leading cause of people living with years of disability worldwide. Notably, thunder-fire moxibustion (TFM) is a new type of moxibustion, which has been widely applied to treat pain syndromes for thousands of years. This study aims to provide evidence to evaluate the effect and safety of TFM in treating LBP.

**Methods:**

A systematic search of PubMed, Web of Science, the Cochrane Library, Embase, EBSCO, CNKI, Wanfang Data, CBM, and VIP (until April 2021) was used to identify studies reporting pain intensity, disability, Japanese Orthopedic Association (JOA) score, and quality of life in patients with LBP. Randomized controlled trials (RCTs), which compared TFM and other therapies in LBP, were included. Meanwhile, methodological quality was evaluated using the Cochrane criteria for risk of bias, and the level of evidence was rated utilizing the GRADE approach.

**Results:**

Twenty-one RCTs, including 2198 patients, satisfied the inclusion criteria. Compared with other therapies, the effect of TFM was statistically significant, pain intensity decreased (SMD = 0.94; 95% CI (0.74, 1.14); *p* < 0.00001), disability improved (SMD = 1.39; 95% CI (0.19, 2.59); *p*=0.02), and the JOA score increased (SMD = −1.34; 95% CI (−1.88, −0.80); *p* < 0.00001). It was also reported that the patient's quality of life improved after treatment for a period of 4 weeks (SMD = −0.29; 95% CI (−0.42, −0.16); *p* < 0.0001) and after a follow-up of 1 month (SMD = −0.20; 95% CI (−0.34, −0.07); *p*=0.003). The evidence level of the results was determined to be very low to low.

**Conclusions:**

Based on the existing evidence, it can be concluded that TFM may have a better effect than other treatments on LBP. However, it is not yet possible to assess the safety level of TFM therapy. Due to the universal low quality of the eligible trials and low evidence level, rigorously designed large-scale RCTs must be conducted in order to further confirm the results in this review.

## 1. Introduction

Low back pain (LBP) is a symptom, not a disease, which results from several different known or unknown abnormalities or diseases [1]. LBP is the most common musculoskeletal health problem with the highest prevalence in the adult population [2]. The estimated lifetime prevalence of LBP is up to 80%, meaning many adults will experience an episode of LBP at least once [3]. According to the Global Burden of Disease Study 2017, LBP was classified as the leading cause of years lived with disability (YLDs) globally. Specifically speaking, the global YLDs for LBP were 42.5 million in 1990 and increased 52.7% to 64.9 million in 2017 [1, 4, 5]. Notably, annual healthcare costs attributed to LBP in the United States are estimated to be $100 billion. Particularly, two-thirds of which were indirect costs of lost wages and productivity, which imposes an economic burden on the healthcare system [6, 7]. Disability and costs attributed to LBP are projected to increase in the coming decades, and this is particularly true in low-income and middle-income countries [1].

Clinicians and researchers have used conventional drugs and surgery to treat LBP for many years, but a large proportion of patients still continue to suffer from LBP [8, 9]. The most commonly used method to relieve pain syndrome of LBP is a nonsteroidal anti-inflammatory drug (NSAID), but it should be noted that their long-term use may increase gastrointestinal and renal risks [6, 10]. At best, surgery has a minimal impact on LBP, which is also more costly and carries a greater risk of adverse effects than nonsurgical management [11]. Throughout the past three decades, changes have been made to critical recommendations in national clinical practice guidelines in Denmark, the United States, and the UK [6, 12, 13]. Greater emphasis is now placed on self-management, physical and psychological therapies, and some forms of complementary medicine, and less emphasis has been placed on pharmacological and surgical treatments.

According to the guidelines of the American College of Physicians, nonpharmacological treatment of superficial heat is recommended for patients who suffer from low back pain (moderate-quality evidence) [6]. As one of the most complementary therapies for LBP, moxibustion is a traditional Chinese medicine (TCM) therapy that has a history of thousands of years in China. Specifically speaking, moxibustion refers to igniting moxa velvet or sticks and then burning or fumigating them on corresponding acupuncture points to prevent and treat diseases by means of heat or medicine [14]. As a new type of moxibustion therapy, thunder-fire moxibustion (TFM) was ameliorated by Prof. Zhao Shibi based on her decades of medical practice experience [15]. Notably, it is widely applied in China to treat diseases such as eye diseases, otolaryngological diseases, osteoarthropathy, gynecological diseases, and pain caused by any other disease [16–20]. Notably, TFM was listed as a critical new technology promotion project by the State Administration of Traditional Chinese Medicine in 2010 [21]. Compared with conventional moxa sticks, TFM has a larger diameter of not only moxa but also agarwood, frankincense, woody, dried ginger, and other TCM [22]. The temperature of TFM can reach up to 240°C when burning, and its average temperature is 142°C higher than that of ordinary moxibustion. Moreover, its warm stimulation involves the epidermis and affects the subcutaneous and muscle layers. When TFM burns, near-infrared rays can also penetrate the deep tissues of the human body, and the penetration depth is more than 10 mm, while conventional moxibustion is about 10 mm [23, 24]. Additionally, TFM can be combined with various manipulations to improve the curative effect, such as pecking and rotating, and arrays can also be used.

Although several clinical trials have been conducted on TFM for treating LBP, based on our understanding, no systematic review and meta-analysis of TFM or TFM combined with other treatments for treating LBP have been reported. Consequently, the aim of this study focused on evaluating the quality of these randomized controlled trials (RCTs) to assess the effect and safety of TFM in treating LBP and better guide clinicians.

## 2. Methods

This meta-analysis was performed according to the Preferred Reporting Items for Systematic Review and Meta-Analyses (PRISMA) guidelines [25].

### 2.1. Data Sources

A systematic literature search was conducted in the following databases from their inception to the period of April 18, 2021: PubMed, Web of Science, the Cochrane Library, Embase, EBSCO, China National Knowledge Infrastructure (CNKI), Wanfang Data, Chinese Science and Technology Periodical Database (VIP), and Chinese Biology Medicine (CBM) disc. The search strategies for PubMed, Embase Cochrane Library, Web of Science, and EBSCO are presented in the Appendix. Other databases were also searched using these terms, but they were slightly modified. Two researchers searched independently and imported the identified literature into EndNote software to delete the duplication and select potential articles by reviewing the titles and abstracts. The full texts of the chosen articles were reviewed according to inclusion and exclusion criteria.

### 2.2. Inclusion Criteria

#### 2.2.1. Types of Studies

All relevant RCTs of TFM for LBP were collected. There were no restrictions on publication type, language, or status.

#### 2.2.2. Types of Participants

Patients with LBP regardless of gender, age, ethnicity, education, and economic status who meet the diagnostic criteria were included in the study [26, 27].

#### 2.2.3. Types of Interventions

The experimental group adopts a single TFM or TFM combined with other therapies. The control group receives other therapies besides TFM, such as usual care, acupuncture, moxibustion, medication, or physical therapy.

#### 2.2.4. Types of Outcome Measures

The outcomes included are pain intensity (including Visual Analogue Scale (VAS) [28]) and disability (on Roland–Morris Disability Questionnaire (RMDQ) [29] and Oswestry Disability Index (ODI) [30]). It should also be noted that other outcomes in this review were the Japanese Orthopedic Association (JOA) score [31] and quality of life (36-item short-form health survey (SF-36) [32]). Two outcome measures were considered to be positive indicators, such as the JOA score and SF-36, while all others were negative indicators. Among the positive indicators, the higher the score, the better the effect of the intervention. On the contrary, among the negative indicators, the lower the score, the better the effect of the intervention.

### 2.3. Data Extraction

A data collection form was created to record selected studies such as the first author, published year, sample size, age, course of the disease, intervention regimens, treatment duration, follow-up duration, and outcomes before extracting the valuable information. Two researchers (Yao and Chen) independently completed the data extraction and the extracted information was reviewed once again upon completion. The divergence of opinion was resolved by consulting the senior reviewer (Sun). If related data were deficient, one researcher (Yao) contacted the writers of the articles for lost information either through telephone or e-mail.

### 2.4. Assessment for Risk of Bias

Two independent reviewers assessed the risk of bias following the Cochrane Handbook for Systematic Reviews of Interventions [33], including the following items: (1) random sequence generation (selection bias), (2) allocation concealment (selection bias), (3) blinding of participants and personnel (performance bias), (4) blinding of outcome assessment (detection bias), (5) incomplete outcome data (attrition bias), (6) selective reporting (reporting bias), and (7) other bias. The evaluation on these items was rated as “low,” “high,” or “unclear.” Meanwhile, an Egger's test could be applied to appraise the extent of publication bias. Divergences were resolved by discussion. If the two investigators were unable to reach an agreement, the third and fourth reviewers (Sun and Du) were consulted for a final decision.

### 2.5. Data Synthesis and Analysis

The meta-analysis was implemented by using RevMan 5.3 (available from the website: https://community.cochrane.org/tools/review-production-tools/revman-5). Change values evaluated efficacy from baseline to endpoint data on each outcome in this meta-analysis [34]. In terms of parallel trials, net changes in measurements (change scores) for the trials were calculated by subtracting the postintervention data from the baseline value. For crossover studies, it was recommended that paired *t*-test data were extracted, which separately evaluated the value of “measurement on intervention” minus “measurement on control” for each participant. However, because this type of data was rarely provided, we resorted to using mean and SD [35]. If SDs were not reported directly, it was calculated from SEM or 95% CI using the following formulas: (1) SD = SEM × $\sqrt{n}$; (2) SD = (upperlimit − lowerlimit) ×  $\sqrt{n}$ ÷ 3.92, where n represents the number of subjects. Change-from-baseline SD was estimated using the equation: (3) SD_change_ = $\sqrt{{\text{SD}}_{\text{baseline}}^{2}+{\text{SD}}_{\text{final}}^{2}-2\times R\times {\text{SD}}_{\text{baseline}}^{2}\times {\text{SD}}_{\text{final}}^{2}}$, where *R* is the correlation coefficient. Through a conservative estimate, a minimum correlation coefficient of 0.5 was used [36]. Notably, *χ*^2^ test and *I*^2^ test were used to measure the heterogeneity among studies. A fixed-effect model was adopted if *I*^2^ < 50% and *P* > 0.1; otherwise, a random-effects model was employed. Dichotomous outcomes were reported as risk ratio (RR) and continuous data as weighted mean difference (WMD) and standard mean difference (SMD). Additionally, we conducted metaregression and subgroup analysis to explore the source of heterogeneity [37]. Sensitivity analysis was performed to evaluate the stability of analysis using different effects models and examining the effects of individual factors on the overall combined effect size. The potential publication bias was tested by employing an inverted funnel chart developed by Egger (Egger's test) when the number of eligible RCTs was more than 10 [38]. The sensitivity analysis and the Egger's test were carried out by STATA 12.0 software (Stata Corp, College Station, TX, USA).

### 2.6. Level of Evidence

The level of evidence was evaluated with the help of the Grading of Recommendations, Assessment, Development, and Evaluation (GRADE) [39]. The level of evidence from low to high was classified into four grades: very low, low, moderate, and high. Particularly, RCTs started with a high level of evidence. Then, the level of evidence was lowered gradually from the five aspects, including risk of bias, indirectness, inconsistency, imprecision, and publication bias. On the contrary, the level of evidence was gradually derived from three factors, which were dose-response gradient, large effect, and plausible confounding.

## 3. Results

### 3.1. Study Selection

**Figure 1:**
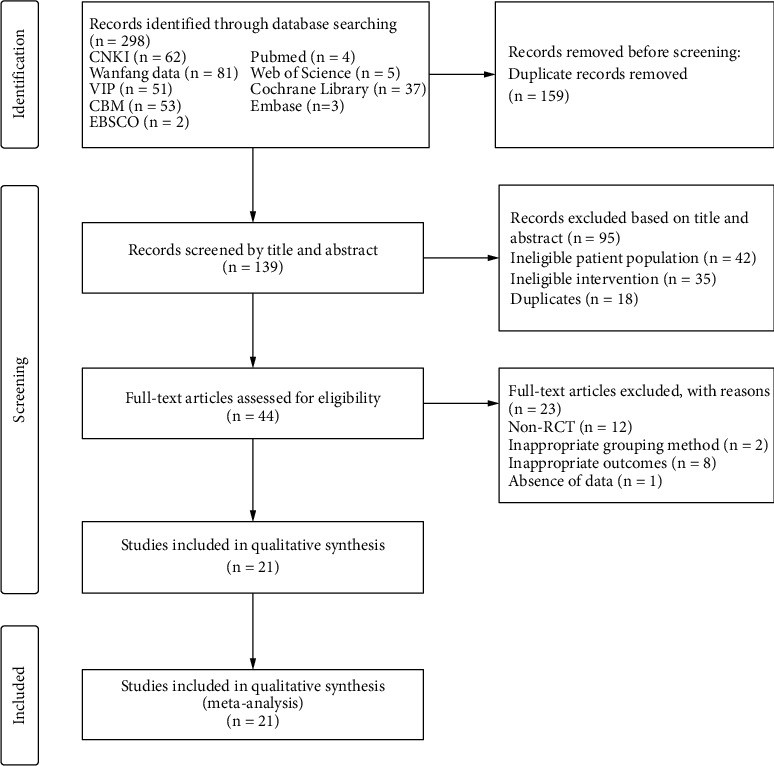
Flowchart of the study selection process.

A total of 298 potential studies were identified through initial database searching. One hundred and fifty-nine articles were deleted due to duplication. After reviewing the titles and abstracts, 95 studies were excluded because of ineligible patient populations (*n* = 42), ineligible intervention (*n* = 35), and duplicates (*n* = 18). Then, the eligibility of the remaining 44 studies was evaluated by reviewing the full text. Particularly, 23 studies were excluded due to non-RCT (*n* = 12), inappropriate grouping method (*n* = 2), and the absence of data (*n* = 1). Finally, a total of 21 RCTs [40–60] satisfied the inclusion criteria and were included in the systematic review. The selection process and reasons for exclusion are shown in Figure 1.

### 3.2. Study Characteristics

All included trials were conducted in China and published from the period of 2011 to 2021. One article [53] was published in English, and twenty were published in Chinese. One RCT adopted a 3-arm parallel-group design [45], and 20 trials used a 2-arm parallel-group design. Sample sizes varied from 53 to 420 participants, and a total of 2198 patients were included. Eighteen RCTs [41–45, 48–57, 59, 60] used VAS to assess pain intensity. Meanwhile, three RCTs [46, 48, 50] selected ODI, and one RCT [47] used both ODI and RMDQ to assess disability. Notably, two RCTs [41, 53] adopted SF-36 to assess quality of life, and seven RCTs [40, 43, 48, 49, 55, 57, 58] reported a JOA score.  Table 1 lists the details and characteristics of the included RCTs.

### 3.3. Risk of Bias

**Figure 2:**
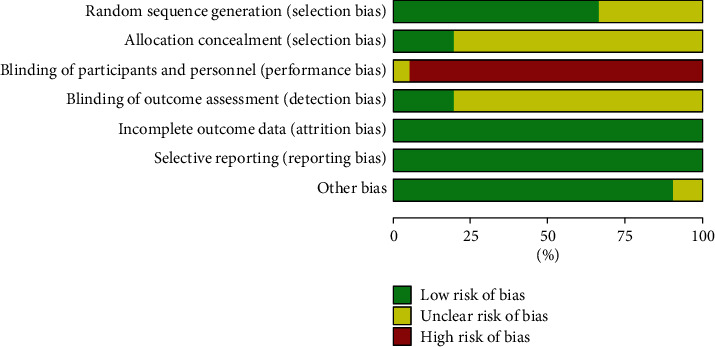
Overall risk of bias analysis of included studies.

**Figure 3:**
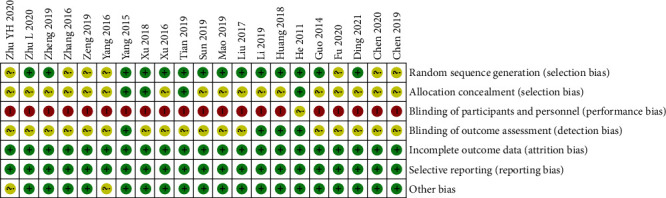
Risk of bias analysis of each included studies.

Based on Cochrane criteria, the risk of bias assessment is shown in Figures 2 and 3. Fourteen [40, 41, 46, 48–55, 58–60] of all 21 studies used a random table for randomization, and the remaining seven trials [42–45, 47, 56, 57] did not provide the methods of sequence generation. Only four trials [50, 53, 58, 60] reported using sequential numbering and opaque sealed envelopes to conduct allocation concealment and the remainder did not provide concealment methods. Although both groups in a study [60] used moxibustion boxes to compare the effects of thunder-fire moxibustion and pure moxibustion and avoided revealing relevant grouping and treatment information to the subjects, the author was not able to ensure the reliability of the blinding method. Consequently, this study is judged to be unclear, and the rest are considered high risk. Four trials [46, 52, 58, 60] reported employing the blindness of the assessor. Two studies [44, 56] only stated that the baseline was not statistically significant but failed to present specific data.

### 3.4. Metaregression

A pooled analysis of improvement was conducted in the pain intensity with TFM treatment using the meta-analysis method. Severe heterogeneity was detected among studies (I^2^ = 75%, c^2^ = 67.47, df = 17, *p* < 0.00001), which demonstrates that it was necessary to conduct the metaregression. Particularly, the metaregression was employed to identify the heterogeneity factor from the possible factors (such as treatment duration, moxibustion method, combined use, and sample size) that may cause heterogeneity. The regression results illustrated that the moxibustion method was the source of heterogeneity *p*=0.032(*p*=0.032). Therefore, a subgroup analysis was employed based on the moxibustion method (array or manipulation).

### 3.5. Results of Meta-Analysis

#### 3.5.1. Pain Intensity

**Figure 4:**
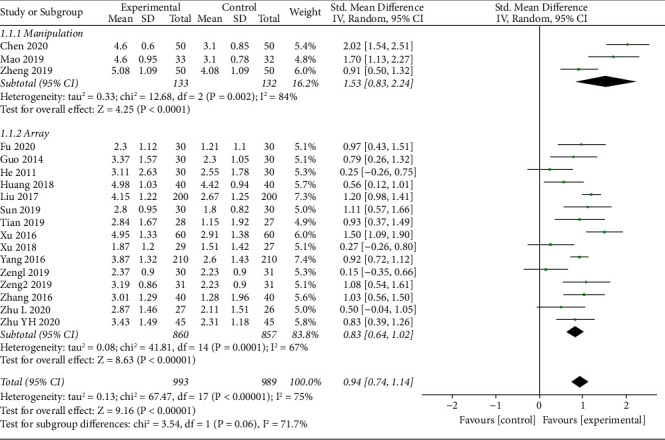
Forest plots of pain intensity.

The forest plot illustrating the results of the meta-analysis for pain intensity is shown in Figure 4. The pain intensity was reported in eighteen studies [41–45, 48–57, 59, 60] with 993 participants in the experimental groups and 989 in the control groups to evaluate the curative effect of TFM. All of these eighteen studies applied the Visual Analogue Scale as the outcome measurements. Despite the use of manipulation or array, the result indicated that TFM was able to significantly reduce pain compared with the control group on LBP (SMD = 0.94, 95% CI (0.74, 1.14), *p* < 0.00001*p* < 0.00001). The subgroup differences test indicated no potential differences between the manipulation group and the array group.

#### 3.5.2. Disability

**Figure 5:**
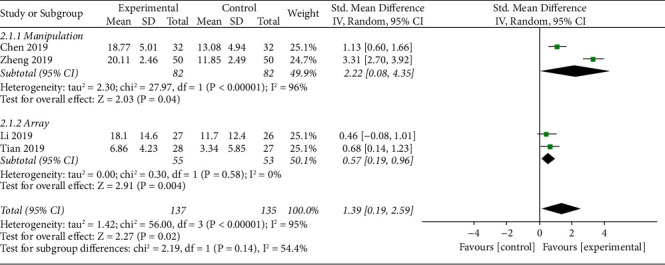
Forest plots of disability.

The forest plot illustrating the results of the meta-analysis for the disability is shown in Figure 5. Four studies measured the level of disability [46–48, 50], they all utilized an Oswestry Disability Index, and one simultaneously used a Roland–Morris Disability Questionnaire [47]. Therefore, the latest data were not used. In total, the level of disability was assessed in 272 participants. Pooled analysis of all trials demonstrated statistically significant improvements in the level of disability in the TFM group compared to the control group (SMD = 1.39, 95% CI (0.19, 2.59), *p*=0.02*p*=0.02). Similarly, a subgroup analysis of different moxibustion methods was conducted and found no statistical difference in improving disability between the manipulation and array groups.

#### 3.5.3. JOA Score

**Figure 6:**
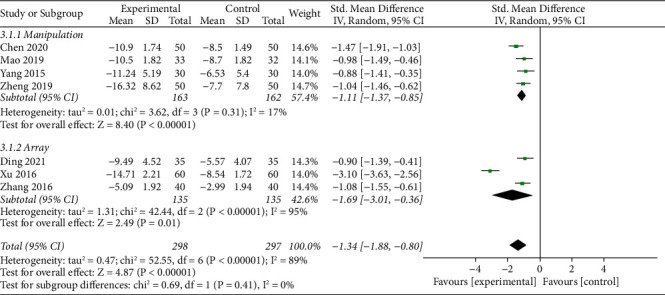
Forest plots of JOA score.

The forest plot illustrating the results of the meta-analysis for the JOA score is shown in Figure 6. There were 7 RCTs [40, 43, 48, 49, 55, 57, 58] using the JOA score to measure the effects for improving LBP. Notably, 595 participants with LBP were involved in the 7 RCTs. All of subgroup analysis results indicated favourable effects of TFM: manipulation group [43, 48, 49, 58] (SMD = −1.11, 95% CI (−1.37, −0.85), *p* < 0.00001*p* < 0.00001) and array group [40, 55, 57] (SMD = −1.69, 95% CI (−3.01, −0.36), *p*=0.01*p*=0.01).

#### 3.5.4. Quality of Life

**Figure 7:**
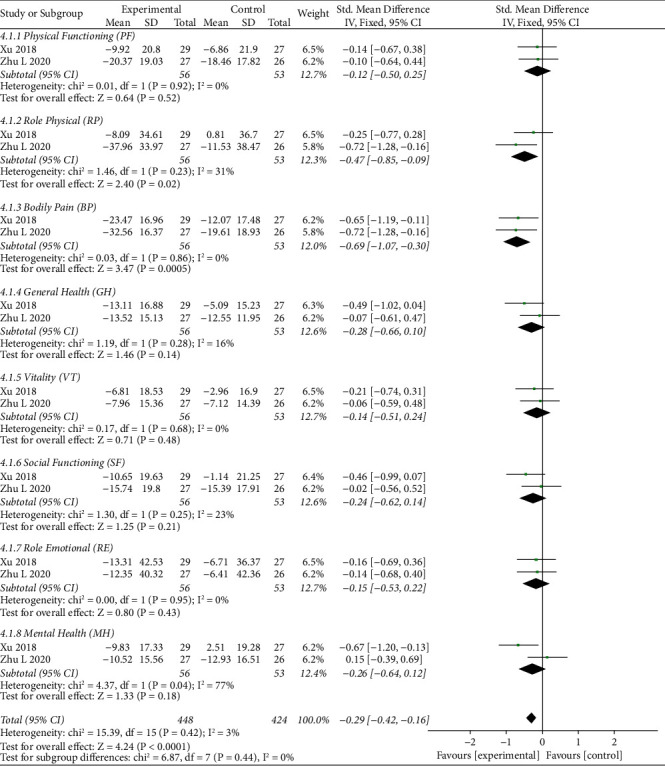
Forest plots of quality of life (for a period of 4 weeks).

**Figure 8:**
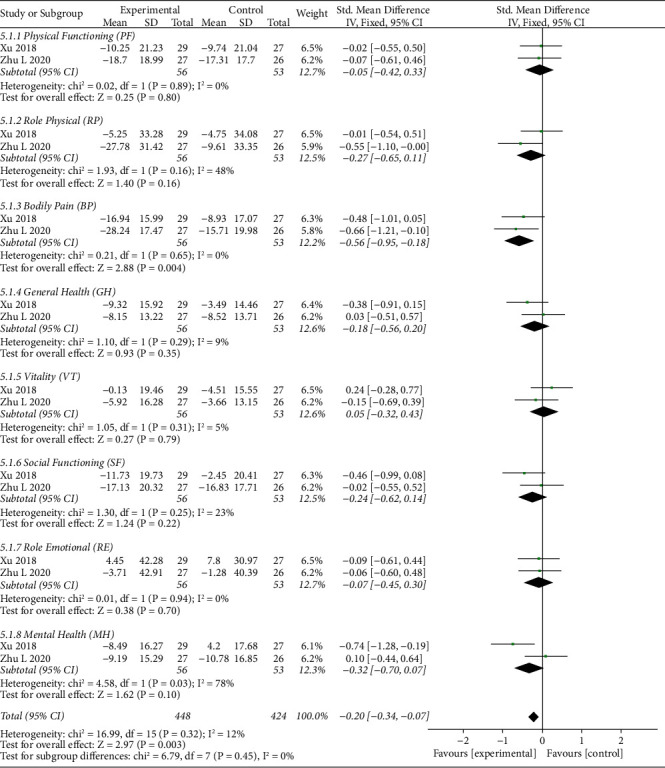
Forest plots of quality of life (after a follow-up of 1 month).

The forest plot illustrating the results of the meta-analysis for the quality of life is shown in Figures 7 and 8. There were two RCTs [41, 53] that adopted SF-36 as an outcome to assess quality of life. The SF-36 contains eight domains: physical functioning (PF), role physical (RP), bodily pain (BP), general health (GH), vitality (VT), social functioning (SF), role emotional (RE), and mental health (MH). In general, significant improvement was found with thunder-fire moxibustion compared with the control group after treatment for a period of 4 weeks (SMD = −0.29, 95% CI (−0.42, −0.16), *p* < 0.0001*p* < 0.0001) and after a 1-month follow-up (SMD = −0.20, 95% CI (−0.34, −0.07), *p*=0.003*p*=0.003). After treatment for 4 weeks and upon assessing the singular domain of the SF-36, TFM was associated with significantly better scores in RP (SMD = −0.47, 95% CI (−0.85, −0.09), *p*=0.02*p*=0.02) and BP (SMD = -0.69, 95% CI (−1.07, −0.30), *p*=0.0005*p*=0.0005). There were no stark differences in the other factors, which indicate no obvious difference between the TFM group and the control group in terms of PF (SMD = −0.12, 95% CI (−0.50, 0.25), *p*=0.52*p*=0.52), GH (SMD = −0.28, 95% CI (−0.66, 0.10), *p*=0.14*p*=0.14), VT (SMD = −0.14, 95% CI (−0.51, 0.24), *p*=0.48*p*=0.48), SF (SMD = −0.24, 95% CI (−0.62, 0.14), *p*=0.21*p*=0.21), RE (SMD = −0.15, 95% CI (−0.53, 0.22), *p*=0.43*p*=0.43), and MH (SMD = −0.26, 95% CI (−0.64, 0.12), *p*=0.18*p*=0.18). After a 1-month follow-up, the TFM group had a significant effect compared with the control group only in terms of BP (SMD = −0.56, 95% CI (−0.95, −0.18), *p*=0.004*p*=0.004). However, there is no statistical difference between the two groups in the domain of PF (SMD = −0.05, 95% CI (−0.42, 0.33), *p*=0.80*p*=0.80), RP (SMD = −0.27, 95% CI (−0.65, 0.11), *p*=0.16*p*=0.16), GH (SMD = −0.18, 95% CI (−0.56, 0.20), *p*=0.35), VT (SMD = 0.05, 95% CI (−0.32, 0.43), *p*=0.79*p*=0.79), SF (SMD = −0.24, 95% CI (−0.62, 0.14), *p*=0.22*p*=0.22), RE (SMD = −0.07, 95% CI (−0.45, 0.30), *p*=0.70*p*=0.70), and MH (SMD = −0.32, 95% CI (−0.70, 0.07), *p*=0.10*p*=0.10).

### 3.6. Adverse Events

Adverse events reported in the studies were sparse. Of the included 21 studies, seven studies [40, 50, 52, 57–59] mentioned the term “adverse events,” of which six studies [50, 52, 57–60] only descriptively reported that no adverse reaction occurred in either the test or control groups. Ding [40] reported that two patients in the control group experienced symptoms such as dizziness and fatigue at the initial stage of treatment. Two patients in the experimental group had slight redness and a miliary rash on local skin, which was relieved after approximately two days. Besides such symptoms, there were no other uncomfortable reactions in the two groups. It should be noted that the adverse events of the two groups were tolerable and did not require specific interventions.

### 3.7. TFM Performed for LBP

The selection of acupoints was also assessed for the included researches. A total of 15 acupoints were selected from 21 studies. Two studies [50, 53] selected the same acupoint therapy, and two other studies [59, 60] selected another similar acupoint therapy. Meanwhile, four other studies [47, 51, 54, 56] only chose the Ashi point. Apart from that, the remaining studies were different. It was observed that BL23 (14 studies [40–42, 44–46, 48–50, 52, 53, 57, 59, 60], 66.7%) had the highest frequency of use, followed by Ashi point (11 studies [42–44, 47, 51, 52, 54, 56, 58–60], 52.4%), GV3 (9 studies [40, 41, 45, 46, 48, 50, 52, 53, 57], 42.9%), BL40 (7 studies [42, 44, 45, 48, 49, 55, 58], 33.3%), GV4 (6 studies [40, 41, 46, 50, 52, 53], 28.6%), GB30/EX-B2 (5 studies [42, 43, 45, 48, 49, 52, 55, 57, 58], 23.8%), and BL20/BL25 (3 studies [41, 45, 46, 50, 53], 14.3%). Notably, the other acupoints were utilized only one time, which are listed in Table 2.

### 3.8. Sensitivity Analysis

**Figure 9:**
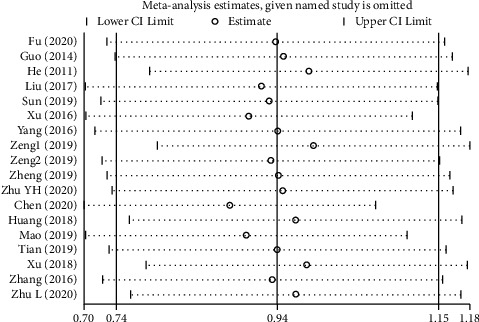
Sensitivity analysis of the pain intensity.

Sensitivity analysis was carried out as a means to evaluate the stability of meta-analysis by using STATA 12.0 software, such as pain intensity (Figure 9). After the sequential exclusion of individual studies one by one, the WMDs were recalculated to identify any significant change in our results. Sensitivity analysis showed that the exclusion of any single study was unlikely to overturn our findings.

### 3.9. Publication Bias

**Figure 10:**
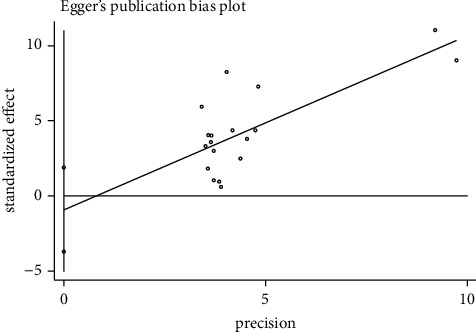
Regression diagram of Egger's test based on pain intensity.

Based on the pain intensity of the STATA 12.0 software, publication bias was analysed through Egger's test, which is shown in Figure 10. The results demonstrated a *p* value of 0.504. This is more significant than 0.05 and reflected no publication bias (from a statistical significance perspective) for this present meta-analysis.

### 3.10. Level of Evidence

The results of GRADE analysis revealed that the evidence quality of all outcome indicators was determined to be low or very low, which was not conducive to our result recommendation. As listed in Table 3, we lowered the levels mainly by the risk of bias, imprecision, and inconsistency.

## 4. Discussion

We intend to appraise the curative effect and the safety of TFM on LBP. About 21 RCTs were included for meta-analysis after searching and screening the major domestic and foreign databases by evidence-based medicine. The result revealed that TFM had favourable effects for LBP in comparison with TFM and other active treatments or TFM combined with other active treatments with active treatments alone. Notably, TFM can relieve pain and disability caused by LBP. It should also be noted that in terms of the JOA score, TFM had favourable effects for LBP in the comparison of TFM and other active treatments or TFM combined with other active treatments with active treatments alone. Particularly, TFM significantly improved the quality of life in the RP and BP dimensions compared to the control group after a 4-week treatment. Meanwhile, it only improved in the BP dimension relative to the control group after a follow-up of 1 month. We recommend the effect of TFM in LBP because of the low-to-very low level of evidence.

The curative effect of TFM is closely related to moxibustion methods, and there are various methods that are suitable for different diseases. The moxibustion methods of TFM include manipulations (such as bird pecking, circling, and spiral moxibustion) and array method. Specifically speaking, the array method refers to the use of single, double, or multihole moxibustion boxes. Based on the condition of different patients, two or more moxibustion boxes are placed on the patients in horizontal array, vertical array, oblique array, T-shaped array, etc. Notably, the majority of articles included in this study used the array method. Through the strong thermal stimulation of the moxibustion stick burning, the array method gathers the heat and expands the heated area, which increases homogeneity to a certain extent. Impressively, our regression analysis also verified this result. Here, we used the treatment duration, the moxibustion method, combined use, and sample size as possible factors for regression analysis and identified that subgroup analysis based on the moxibustion method explained some heterogeneity sources.

Pain intensity, disability, and JOA score of LBP were statistically significant with substantial heterogeneity. As heterogeneity across studies is expected in meta-analyses [61], it is not surprising that there was considerable heterogeneity in the effect of TFM on the LBP. Although a subgroup analysis was performed based on the regression results, heterogeneity still existed in these comparisons. The variety of acupoint selection schemes, treatment frequencies, and courses may have caused unresolved heterogeneity. Specifically speaking, the frequency is usually once a day, but it also includes every other day and once every three days. Intervention time also varied from 15 min to 60 min. It should be noted that these conditions may be related to the cause and duration of LBP.

TFM has unique thermal and infrared effects during burning so it may produce various adverse effects, such as burn wounds, blister, and pruritus [62]. Seven of the 21 studies mentioned adverse events, and only 2 cases experienced local skin redness and miliary rash, which was related to TFM. Moreover, neither of these two patients requires particular medical intervention. Nevertheless, the safety of TFM cannot be definitively concluded due to a relative lack of studies providing details of the adverse events. However, the issue of whether moxibustion-induced burns are actually considered an adverse event still remains controversial [63]. Traditional Chinese moxibustion is also known as scarring moxibustion. It has long been taken for granted that it causes minor burns, scarring, and purulence during treatment, as various ingredients enter the body through burn-damaged skin [64].

Due to the following limitations, we were unable to reach an exact conclusion regarding the effect of TFM. This is especially attributed to the fact that the methodological quality of inclusive studies was low and that there was no multicenter study, and the outcome indicators were subjective. Additionally, the sample size of most studies was small and an inappropriate random method was used. Moreover, there was allocation concealment and a lack of blinding of most studies, which exaggerated the results of the outcome measures. In this study, the correct reporting of allocation concealment and blinding of outcome measurers were both 19.05% of the literature. The blinding of participants and subjects was not successfully performed due to the particularity of the TFM treatment, which could lead to overestimation.

The potential mechanism of TFM for LBP is not yet distinct, but it does have a positive therapeutic effect. Compared to thermal therapy, TFM is based on the TCM meridian theory. Specifically, it uses the heat, thermal infrared radiation, and physicochemical factors produced by drug combustion through meridian and acupoints feeling in achieving WenTong meridian and adjusting human body's energy to treat disease [53]. WenTong meridian means promoting the dredging function of meridians by warming. Chen [65] reported that TFM had an anti-inflammatory effect on model rats with knee osteoarthritis. Its therapeutic mechanism may be related to reducing the contents of TNF-*α* and IL-1*β* in the serum of model rats. Notably, some studies even demonstrated that TNF-a and IL-1*β* seemed to play a significant role in patients suffering from LBP [66, 67]. However, these theories have not yet been fully established. Consequently, there is still a great distance to go before the mechanism involved with TFM is fully understood.

In TCM theory, the most commonly used acupoints for LBP were located in the bladder, gallbladder meridian, and the governor vessel—all of which pass through the waist. In our statistical results of acupoints, the vast majority of acupoints were located on these three meridians. According to textbooks and clinical practice, the acupoints of BL23, BL25, BL40, GV3, GV4, and GB30 were globally used to treat nonspecific and chronic LBP, as reported by Yuan et al. [68, 69]. In addition, Yuan reported that Ashi acupoints are usually reported from all sources. The above statements are consistent with our research results. This illustrated that when the interveners used TCM therapy to treat LBP, such as acupuncture and moxibustion, they followed the TCM theories in selecting acupoints.

This review presented several limitations. First of all, we collected a significant amount of literature through a comprehensive search strategy of nine different databases, without any language restrictions. However, only articles published in Chinese and English were retrieved and all the studies were conducted in China. This may be due to the facts that thunder-fire moxibustion belongs to a category of TCM and that less foreign studies were found in this area. Second, given that the methodological quality of most qualified trials was low, it may lead to serious selection performance and detection bias.

To some extent, this weakened the authenticity and reliability of the evidence for TFM treatment of LBP in this study. Third, although some sources of heterogeneity were identified through regression and subgroup analysis, significant heterogeneity still existed among studies. Finally, the course of TFM was short term (less than 12 weeks) among the included studies so it is unclear whether the long-term practice of TFM is beneficial for LBP patients.

While this systematic review and meta-analysis had some limitations, it nonetheless demonstrated some glaring advantages. Although an increasing number of studies reported TFM to successfully treat LBP patients ranging from case report studies to cohort studies to RCTs, there was no systematic review. This is especially in those that primarily referred to its effectiveness in treating LBP. Hence, this meta-analysis was designed to evaluate the efficacy of TFM for LBP. In addition, we conducted this systematic review and meta-analysis in strict accordance with the PRISMA guidelines, and the content met the criterion. Therefore, we speculated that the results of this review could provide evidence on the efficiency and safety of TFM in treating LBP, which would benefit both patients as well as practitioners.

## 5. Conclusion

This review provided a comprehensive assessment of the quality of the methodology and the level of evidence. Existing evidence indicates that TFM is able to effectively treat LBP. However, the findings should be cautiously interpreted because of universally low-quality eligible trials and low evidence level. The safety of TFM cannot be definitively concluded due to a relative lack of studies that provide details of its adverse effects. In the future, more well-designed, rigorous, large sample, and multicenter prospective randomized controlled trials are needed on this subject to confirm the validity of the results.

## Figures and Tables

**Table 1:** Basic characteristics of eligible RCTs.

Study (author/year)	Number ofparticipants, E/C	Completionnumber, E/C	Mean age(years)	Course of disease(months)	Intervention		Treatment duration	Outcomes	Follow-up
					Experimental group	Control group			

Ding (2021)	35/35	35/35	E: 47.71 ± 10.27 C: 49.26 ± 10.65	E: 3.89 ± 1.45 C: 3.54 ± 1.12	TFM + TCM + Pelvic traction	TCM + Pelvic traction	8 weeks, NR	JOA	NR
Zhu L (2020)	30/30	27/26	E: 66 ± 8 C: 64 ± 7	E: 11.4 ± 5.4 C: 12.1 ± 5.6	TFM + Vibration training	Vibration training	4 weeks, 30 min/QOD	VAS, SF-36	4 weeks
Fu (2020)	30/30	30/30	E: 34.9 ± 2.4 C: 34.6 ± 2.1	NR	TFM + Usual nursing	Usual nursing	2 weeks, 20 min/d	VAS	NR
Chen (2020)	50/50	50/50	E: 41.6 ± 6.7 C: 42.4 ± 6.5	E: 20.8 ± 7.8 C: 20.1 ± 7.3	TFM + Tuina	Tuina	4 weeks, 30–45 min/d	VAS, JOA	NR
Zhu YH (2020)	45/45	45/45	E:>40 C:>40	NR	TFM	Moxibustion	1 week, 40 min/d	VAS	NR
Zeng (2019)	30/31/31	30/31/31	E1: 44.47 ± 8.10 E2: 44.58 ± 8.01 C: 43.36 ± 8.98	NR	E1: TFM E2: TFM + TCM	TCM	10 d, 40–45 min/d	VAS	NR
Li (2019)	30/30	27/26	E: 66.30 ± 7.80 C: 63.54 ± 7.08	NR	TFM + Vibration training	Vibration training	4 weeks, 30 min/QOD	ODI	4 weeks
Chen (2019)	32/32	32/32	E: 36.45 ± 10.32 C: 36.05 ± 10.29	E: 5.09 ± 1.27 C: 5.54 ± 1.24	TFM + Gymnastics	Gymnastics	1 month, once three days	ODI, RMDQ	NR
Zheng (2019)	50/50	50/50	E: 54.49 ± 8.19 C: 54.26 ± 8.24	E: 3.86 ± 2.91 (yr) C: 3.79 ± 2.88 (yr)	TFM + Spinalmanipulation	Spinal manipulation	2 weeks, 60 min/d	VAS, JOA, ODI	NR
Mao (2019)	33/32	33/32	E: 40.1 ± 9.4 C: 39.2 ± 9.1	E: 23.2 ± 11.6 C: 21.6 ± 9.9	TFM + Tuina	Tuina	1 month, 30–60 min/d	VAS, JOA	3 months
Tian (2019)	30/30	28/27	E: 64.21 ± 6.50 C: 63.59 ± 6.95	E: 21.89 ± 11.89 C: 22.81 ± 12.30	TFM + Usual nursing	Usual nursing	4 weeks, 30 min/QOD	VAS, ODI	1 month
Sun (2019)	30/30	30/30	E: 45.3 ± 3.8 C: 46.0 ± 4.9	E: 23.0 ± 6.2 (d) C: 25.0 ± 4.1 (d)	TFM + Acupuncture	Acupuncture	10 d, 20–30 min/d	VAS	NR
Huang (2018)	40/40	40/40	E: 39.30 ± 5.10 C: 40.40 ± 5.20	E: 5.36 ± 0.65 (yr) C: 5.60 ± 0.50 (yr)	TFM + Tuina + Scrapping	Tuina + Scrapping	20 d, 20 min/d	VAS	NR
Xu (2018)	32/31	29/27	E: 65.16 ± 6.82 C: 63.90 ± 7.59	E: 11.31 ± 4.03 C: 11.32 ± 4.16	TFM + Drug therapy	Drug therapy	4 weeks, 30 min/QOD	VAS, SF-36	1 month
Liu (2017)	200/200	200/200	E: 40.93 ± 6.22 C: 41.45 ± 5.97	E: 1.02 ± 0.56 (d) C: 1.12 ± 0.59 (d)	TFM + Acupuncture	Acupuncture	2 weeks, 20 min/d	VAS	NR
Xu (2016)	60/60	60/60	E: 43.10 ± 5.30 C: 42.70 ± 5.30	E: 3.20 ± 0.50 (yr) C: 3.10 ± 0.50 (yr)	TFM + Acupuncture + Tuina + Usual nursing	Acupuncture + Tuina + Usual nursing	3 d, 20–30 min/d	VAS, JOA	NR
Yang (2016)	210/210	210/210	44.5 ± 9.0	<2 weeks	TFM + Conventional drug therapy	Conventional drug therapy	2 weeks, 30 min/d	VAS	NR
Zhang (2016)	40/40	40/40	E: 53.47 ± 10.45 C: 51.25 ± 9.75	E: 2.7 ± 1.6 (yr) C: 2.4 ± 1.8 (yr)	TFM + TCM + Acupuncture	TCM + Acupuncture	2 weeks, 15 min/d	VAS, JOA	1 month
Yang (2015)	30/30	30/30	E: 39.30 ± 16.14 C: 38.40 ± 15.52	E: 8.37 ± 6.52 (yr) C: 8.17 ± 5.72 (yr)	TFM + Acupuncture	Acupuncture	2 weeks, 50–60 min/d	JOA	NR
Guo (2014)	30/30	30/30	E: 48 ± 1.18 C: 49 ± 2.04	E: 10.1 ± 3.98 (yr) C: 11.2 ± 4.04 (yr)	TFM	Moxibustion	1 week, 30 min/d	VAS	NR
He (2011)	30/30	30/30	E: 40.33 ± 9.61 C: 38.30 ± 15.87	E: 5.67 ± 4.39 (yr) C: 4.70 ± 4.92 (yr)	TFM	Moxibustion	1 d, 30 min	VAS	NR

Follow-up	Moxibustion acupoint	Moxibustion method	Adverse events						

NR	BL23, GV3, and GV4	Array	E: 2 cases had slight rednessand miliary rash on local skin. C: 2 cases developeddizziness and fatigue.						
4 weeks	GV3, GV4, BL18, BL23, and BL25	Array	NR						
NR	BL23, BL40, EX-B2, and Ashi point	Array	NR						
NR	ST41, GB39, GB30, GB34, and Ashi point	Manipulation	NR						
NR	BL23, BL40, and Ashi point	Array	NR						
NR	BL23, BL25, BL40, BL60, GB30, GV3, and EX-B2	Array	NR						
4 weeks	BL20, BL23, BL25, GV3, and GV4	Array	NR						
NR	Ashi point	Manipulation	NR						
NR	BL23, BL40, GB30, and GV3	Manipulation	NR						
3 months	BL23, BL26, BL40, and GB30	Manipulation	NR						
1 month	BL20, BL23, GV3, and GV4	Array	No adverse events occurred.						
NR	Ashi point	Array	NR						
NR	BL23, GV3, GV4, EX-B2, and Ashi point	Array	No adverse events occurred.						
1 month	BL20, BL23, GV3, and GV4	Array	NR						
NR	Ashi point	Array	NR						
NR	BL40 and EX-B2	Array	NR						
NR	Ashi point	Array	NR						
1 month	BL23, GV3, and EX-B2	Array	No adverse events occurred.						
NR	BL40, GB30, and Ashi point	Manipulation	No adverse events occurred.						
NR	BL23 and Ashi point	Array	No adverse events occurred.						
NR	BL23 and Ashi point	Array	No adverse events occurred.						

E: experimental group; C: control group; d: day; yr: year; TFM: thunder-fire moxibustion; TCM: traditional Chinese medicine; NR: not reported; VAS: Visual Analogue Scale; JOA: Japanese Orthopedic Association score; ODI: Oswestry Disability Index; SF-36: 36-item short-form health survey; RMDQ: Roland–Morris Dysfunction Questionnaire.

**Table 2:** The most frequently used acupoint.

Order	Acupoints	Frequency (%, *N* = 21)
1	BL23	14 (66.7%)
2	Ashi point	11 (52.4%)
3	GV3	9 (42.9%)
4	BL40	7 (33.3%)
5	GV4	6 (28.6%)
6	GB30/EX-B2	5 (23.8%)
7	BL20/BL25	3 (14.3%)
8	BL18/BL26/BL60/GB34/GB39/ST41	1 (4.8%)

**Table 3:** Level of evidence.

Variable (studies)	Samplesize (E/C)	*I* ^2^ (%)	Riskof bias	Inconsistency	Indirectness	Imprecision	Publicationbias	Effect (95% CI)	Level of evidence
1. Pain intensity									
1.1. Manipulation (3 RCTs)	133/132	84	Serious①	Serious②	Non	Serious③	Non	SMD 1.53 higher (0.83 to 2.24 higher)	⊕○○○ Very low
1.2. Array (15 RCTs)	860/857	67	Serious①	Serious②	Non	Non	Non	SMD 0.83 higher (0.64 to 1.02 higher)	⊕⊕○○ Low
2. Disability									
2.1. Manipulation (2 RCTs)	82/82	96	Serious①	Serious②	Non	Serious③	Non	SMD 2.22 higher (0.08 to 4.35 higher)	⊕○○○ Very low
2.2. Array (2 RCTs)	55/53	0	Serious④	Non	Non	Serious③	Non	SMD 0.57 higher (0.19 to 0.96 higher)	⊕⊕○○ Low
3. JOA score									
3.1. Manipulation (4 RCTs)	163/162	17	Serious①	Non	Non	Serious③	Non	SMD 1.11 lower (1.37 to 0.85 lower)	⊕⊕○○ Low
3.2. Array(3 RCTs)	135/135	95	Serious①	Serious②	Non	Serious③	Non	SMD 1.69 lower (3.01 to 0.36 lower)	⊕○○○ Very low
4. Quality of life									
4.1. 4 weeks (2 RCTs)	56/53	21	Serious④	Non	Non	Serious③	Non	MD 5.36 lower (7.91 to 2.81 lower)	⊕⊕○○ Low
4.2. 8 weeks (2 RCTs)	56/53	19	Serious④	Non	Non	Serious③	Non	MD 3.86 lower (6.37 to 1.36 lower)	⊕⊕○○ Low

E: experimental group; C: control group; CI: confidence interval; RCT: randomized controlled trial; SMD: standard mean difference; MD: mean difference. (1) Blind method is missing, allocation hidden report is insufficient, and random method description is not clear; (2) statistical heterogeneity and clinical heterogeneity were more significant; (3) the total sample size was small; (4) blind method is missing and allocation hidden report is insufficient. ⊕⊕◯◯ represents the low level of evidence. ⊕◯◯◯ represents the very low level of evidence.

## Data Availability

The data supporting the findings of this study are available within the article and its supplementary materials.

## Abbreviations

LBP: Low back pain
TFM: Thunder-fire moxibustion
CBM: Chinese Biomedical Literature Database
CNKI: China National Knowledge Infrastructure
VIP: Chinese Science and Technology Periodical Database
RCTs: Randomized controlled trials
YLDs: Years lived with disability
TCM: Traditional Chinese medicine
PRISMA: Preferred Reporting Items for Systematic Reviews and Meta-Analyses
VAS: Visual Analogue Scale
NRS: Numerical rating scale
RMDQ: Roland–Morris Disability Questionnaire
ODI: Oswestry Disability Index
JOA: Japanese Orthopedic Association score
SF-36: 36-item short-form health survey
PF: Physical functioning
RP: Role physical
BP: Bodily pain
GH: General health
VT: Vitality
SF: Social functioning
RE: Role emotional
MH: Mental health.

## Supplementary Materials

Supplementary Materials PRISMA 2020 Checklist. Appendix search strategies.
