# Supplementary material for: The Effect and Safety of Thunder-Fire Moxibustion for Low Back Pain: A Meta-Analysis of Randomized Controlled Trials
Source: Evid Based Complement Alternat Med. 2022 May 18;2022:6114417. doi: 10.1155/2022/6114417 (PMC9132655; doi:10.1155/2022/6114417)
Supplement: Supplementary Materials — PRISMA 2020 Checklist. Appendix search strategies. [file 6114417.f1.zip › 6114417.f1/Appendix search strategies..docx]

## Appendix: search strategies

*PubMed*

#1. low back pain[Mesh]

#2. ((((((((((((((((((((((((((((((((((((low back pain*[Title/Abstract]) OR lower back pain*[Title/Abstract]) OR back pain*[Title/Abstract]) OR low* back[Title/Abstract]) OR back ache*[Title/Abstract]) OR lumbago[Title/Abstract]) OR lumb?r disc herniation[Title/Abstract]) OR Intervertebral disc disease[Title/Abstract]) OR sciatica[Title/Abstract]) OR LDH[Title/Abstract]) OR LIDH[Title/Abstract]) OR intervertebral disk displacement[Title/Abstract]) OR lumbocrural pain[Title/Abstract]) OR lumb?r spinal stenosis[Title/Abstract]) OR lumb?r muscle strain[Title/Abstract]) OR strain of lumb?r muscles[Title/Abstract]) OR injury of lumb?r muscles[Title/Abstract]) OR low back strain[Title/Abstract]) OR spinal instabilities[Title/Abstract]) OR flatback syndrome[Title/Abstract]) OR back disorder[Title/Abstract]) OR spondylosis[Title/Abstract]) OR coccydynia[Title/Abstract]) OR lumb* pain*[Title/Abstract]) OR dorsalgia[Title/Abstract]) OR lumb?r Vertebrae[Title/Abstract]) OR coccyx[Title/Abstract]) OR intervertebral disc[Title/Abstract]) OR discitis" OR "sacrum[Title/Abstract]) OR intervertebral disc degeneration[Title/Abstract]) OR disc degenerat*[Title/Abstract]) OR disc prolapse*[Title/Abstract]) OR disc herniat*[Title/Abstract]) OR spinal fusion[Title/Abstract]) OR facet joint*[Title/Abstract]) OR postlaminectomy[Title/Abstract]) OR spinal pain[Title/Abstract]

#3. #1 OR #2

#4. (((((thunder fire moxibustion[Title/Abstract]) OR thunder-fire moxibustion[Title/Abstract]) OR lei huo jiu[Title/Abstract]) OR leihuo moxibustion[Title/Abstract] OR thunder-fire needle[Title/Abstract]) OR thunder fire God moxibustion[Title/Abstract]

#5. ((((randomized controlled trial[Title/Abstract]) OR randomised controlled trial[Title/Abstract]) OR random[Title/Abstract]) OR randomly[Title/Abstract]) OR placebo[Title/Abstract]

#6. #3 AND #4 AND #5

*Embase*

#1. low back pain/exp

#2. 'low back pain':ta,ab,kw OR 'lower back pain':ta,ab,kw OR 'back pain*':ta,ab,kw OR 'low* back':ta,ab,kw OR 'lumbago':ta,ab,kw OR 'backache*':ta,ab,kw OR 'lumb?r disc herniation':ta,ab,kw OR 'intervertebral disc disease':ta,ab,kw OR 'sciatica':ta,ab,kw OR 'ldh':ta,ab,kw OR 'lidh':ta,ab,kw OR 'intervertebral disk displacement':ta,ab,kw OR 'lumbocrural pain':ta,ab,kw OR 'lumb?r spinal stenosis':ta,ab,kw OR 'lumb?r muscle strain':ta,ab,kw OR 'strain of lumb?r muscles':ta,ab,kw OR 'injury of lumb?r muscles':ta,ab,kw OR 'low back strain':ta,ab,kw OR 'spinal instabilities':ta,ab,kw OR 'flatback syndrome':ta,ab,kw OR 'back disorder':ta,ab,kw OR 'spondylosis':ta,ab,kw OR 'coccydynia':ta,ab,kw OR 'lumb* pain*':ta,ab,kw OR 'dorsalgia':ta,ab,kw OR 'lumb?r vertebrae':ta,ab,kw OR 'coccyx':ta,ab,kw OR 'intervertebral disc':ta,ab,kw OR 'discitis':ta,ab,kw OR 'sacrum':ta,ab,kw OR 'intervertebral disc degeneration':ta,ab,kw OR 'disc degenerat*':ta,ab,kw OR 'disc prolapse*':ta,ab,kw OR 'disc herniat*':ta,ab,kw OR 'spinal fusion':ta,ab,kw OR 'facet joint*':ta,ab,kw OR 'postlaminectomy':ta,ab,kw OR 'spinal pain':ta,ab,kw

#3. #1 OR #2

#4. 'thunder fire moxibustion':ti,ab,kw OR 'thunder-fire moxibustion':ti,ab,kw OR 'lei huo jiu':ti,ab,kw OR 'leihuo moxibustion':ti,ab,kw OR 'thunder-fire needle':ti,ab,kw OR 'thunder fire god moxibustion':ti,ab,kw

#5. 'randomized controlled trial':ab,ti OR 'randomised controlled trial':ab,ti OR random:ab,ti OR randomly:ab,ti OR placebo:ab,ti

#6. #3 AND #4 AND #5

*Cochrane Library*

#1. MeSH descriptor: [Low Back Pain] this term only

#2. ("low back pain[MeSH]" OR "lower back pain" OR "back pain*" OR "low* back" OR "lumbago" OR "backache*" OR "lumb?r disc herniation" OR "Intervertebral disc disease" OR "sciatica" OR "LDH" OR "LIDH" OR "intervertebral disk displacement" OR "lumbocrural pain" OR "lumb?r spinal stenosis" OR "lumb?r muscle strain" OR "strain of lumb?r muscles" OR "injury of lumb?r muscles" OR "low back strain" OR "spinal instabilities" OR "flatback syndrome" OR "back disorder" OR "spondylosis" OR "coccydynia" OR "lumb* pain*" OR "dorsalgia" OR "lumb?r Vertebrae" OR "coccyx" OR "intervertebral disc" OR "discitis" OR "sacrum" OR "intervertebral disc degeneration" OR "disc degenerat*" OR "disc prolapse*" OR "disc herniat*" OR "spinal fusion" OR "facet joint*" OR "postlaminectomy" OR "spinal pain"):ti,ab,kw

#3. #1 OR #2

#4.("thunder fire moxibustion" OR "thunder-fire moxibustion" OR "lei huo jiu" OR "leihuo moxibustion" OR "thunder fire God moxibustion"):ti,ab,kw

#5. (randomized controlled trials):ti,ab,kw OR (randomised controlled trials):ti,ab,kw OR (random):ti,ab,kw OR (randomly):ti,ab,kw OR (placebo):ti,ab,kw

#6. #3 AND #4 AND #5

*Web of Science*

#1.TS=(“low back pain” OR “lower back pain” OR “back pain*” OR “low* back” OR “lumbago” OR “backache*” OR “lumb?r disc herniation” OR “Intervertebral disc disease” OR “sciatica” OR “LDH” OR “LIDH” OR “intervertebral disk displacement” OR “lumbocrural pain” OR “lumb?r spinal stenosis” OR “lumb?r muscle strain” OR “strain of lumb?r muscles” OR “injury of lumb?r muscles” OR “low back strain” OR “spinal instabilities” OR “flatback syndrome” OR “back disorder” OR “spondylosis” OR “coccydynia” OR “lumb* pain*” OR “dorsalgia” OR “lumb?r Vertebrae” OR “coccyx” OR “intervertebral disc” OR “discitis” OR “sacrum” OR “intervertebral disc degeneration” OR “disc degenerat*” OR “disc prolapse*” OR “disc herniat*” OR “spinal fusion” OR “facet joint*” OR “postlaminectomy” OR “spinal pain”)

#2.TS=(thunder fire moxibustion OR thunder-fire moxibustion OR lei huo jiu OR leihuo moxibustion OR thunder-fire needle OR thunder fire God moxibustion)

#3.TS=(randomized controlled trials OR randomised controlled trials OR random OR randomly OR placebo)

#4. #1 AND #2 AND #3

*EBSCO*

#1.SU low back pain

#2.TX low back pain OR TX lower back pain OR TX back pain* OR TX low* back OR TX lumbago OR TX backache* OR TX lumb?r disc herniation OR TX Intervertebral disc disease OR TX sciatica OR TX LDH OR TX LIDH OR TX intervertebral disk displacement OR TX lumbocrural pain OR TX lumb?r spinal stenosis OR TX lumb?r muscle strain OR TX strain of lumb?r muscles OR TX injury of lumb?r muscles OR TX low back strain OR TX spinal instabilities OR TX flatback syndrome OR TX back disorder OR TX spondylosis OR TX coccydynia OR TX lumb* pain* OR TX dorsalgia OR TX lumb?r Vertebrae OR TX coccyx OR TX intervertebral disc OR TX discitis OR TX sacrum OR TX intervertebral disc degeneration OR TX disc degenerat* OR TX disc prolapse* OR TX disc herniat* OR TX spinal fusion OR TX facet joint* OR TX postlaminectomy OR TX spinal pain

#3. #1 OR #2

#4. TX thunder fire moxibustion OR TX thunder-fire moxibustion OR TX lei huo jiu OR TX leihuo moxibustion OR TX thunder-fire needle OR TX thunder fire God moxibustion

#5. TX randomized controlled trials OR TX randomised controlled trials OR TX random OR TX randomly OR TX placebo

#6. #3 AND #4 AND #5
